# Genomic, Proteomic, Morphological, and Phylogenetic Analyses of vB_EcoP_SU10, a *Podoviridae* Phage with C3 Morphology

**DOI:** 10.1371/journal.pone.0116294

**Published:** 2014-12-31

**Authors:** Mohammadali Khan Mirzaei, Harald Eriksson, Kie Kasuga, Elisabeth Haggård-Ljungquist, Anders S. Nilsson

**Affiliations:** 1 Department of Molecular Biosciences, The Wenner-Gren Institute Stockholm University, SE-10691, Stockholm, Sweden; 2 Cancer Proteomics Mass Spectrometry, Department of Oncology-Pathology, Science for Life Laboratory, Karolinska Institute, Box 1031, SE-17121, Solna, Sweden; Rockefeller University, United States of America

## Abstract

A recently isolated phage, vB_EcoP_SU10 (SU10), with the unusual elongated C3 morphotype, can infect a wide range of *Escherichia coli* strains. We have sequenced the genome of this phage and characterized it further by mass spectrometry based proteomics, transmission electron microscopy (TEM), scanning electron microscopy (SEM), and ultra-thin section electron microscopy. The genome size is 77,327 base pairs and its genes, and genome architecture, show high similarity to the phiEco32 phage genes and genome. The TEM images reveal that SU10 have a quite long tail for being a *Podoviridae* phage, and that the tail also changes conformation upon infection. The ultra-thin section electron microscopy images of phages at the stage of replication within the host cell show that the phages form a honeycomb-like structure under packaging of genomes and assembly of mature capsids. This implies a tight link between the replication and cutting of the concatemeric genome, genome packaging, and capsid assembly. We have also performed a phylogenetic analysis of the structural genes common between *Podoviridae* phages of the C1 and C3 morphotypes. The result shows that the structural genes have coevolved, and that they form two distinct groups linked to their morphotypes. The structural genes of C1 and C3 phages appear to have diverged around 280 million years ago applying a molecular clock calibrated according to the presumed split between the *Escherichia – Salmonella* genera.

## Introduction

Phages are considered to be the most abundant form of life on earth since the number of phages in the biosphere has been estimated to be in the order of 10^31^ [1], [2]. The biodiversity of phages is enormous, because of their high specificity for their bacterial targets, and capability to acquire genes either from their hosts or from other phages by horizontal transfer. Phage genomes can be characterised as mosaics, and it is not uncommon that the genes show a shifting relationship to other phages. The majority of phages belong to the order C*audovirales*, which contains double-stranded DNA phages with a tail. The order is divided into three families; S*iphoviridae* phages have a long flexible tail, M*yoviridae* phages have a complex contractile tail, and *Podoviridae* phages exhibit a very short tail, or no tail at all.

**Figure 1:**
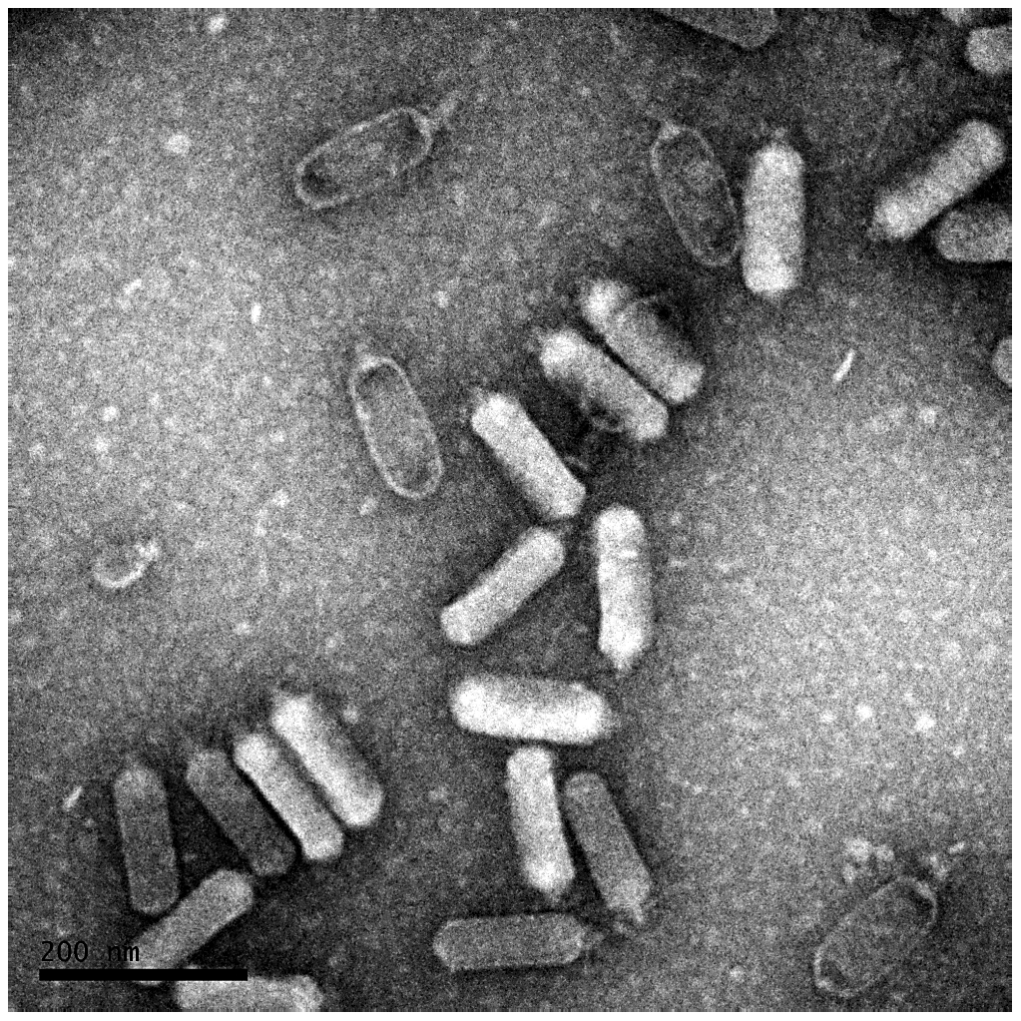
Transmission electron microscopy of negatively stained SU10. Measured head length is 137±4.2 nm.

Thirty virulent phages were isolated from a waste water plant outside Stockholm, Sweden, using the *Escherichia coli* (*E. coli*) reference collection (ECOR) as target bacteria [3]. Six phages were selected for further studies based on their wide host range, growing not only on many ECOR strains but also on extended spectrum beta lactamase encoding (ESBL) *E. coli* strains. Transmission electron microscopy (TEM) studies of the selected phages revealed a phage with the rare C3 morphology of the *Podoviridae* family, which we named vB_EcoP_SU10 or SU10 for short. Phages belonging to this morphotype have been found to infect bacteria belonging to two distantly related groups: Gram-negative enteric bacteria and Gram-positive lactococci [4]. They have an elongated head of about 90–223 nm long, varying in length from phage to phage, and a short tail [5] (Fig. 1). The majority of C3 phages have only been characterized by TEM, but a few have been completely genome sequenced and annotated, and the gene expression of some phages' genes have also been analysed. The *E. coli* phage phiEco32, isolated from the Mtkvari (Kura) river in Tbilisi, Georgia, is the most well characterized member of this group [6]. Other analysed C3 phages growing on Gram negative enterobacteria include the *Serratia marcescens* phage KSP100 isolated in Japan [7], The *Salmonella enterica* Newport phage 7–11 [8], [9] and the *Cronobacter sakazakii* phage vB_CsaP_GAP52 isolated in Canada (NCBI NC_019402.1), and the *E. coli* phage NJ01 isolated in China [10]. The two *E. coli* phages ECMP2 (submitted as KBNP135 in GenBank, NC_018859.1) and KBNP1711 (KM044272) isolated in South Korea, are probably of the C3 morphotype, but this has not been confirmed by TEM [11]. The *Lactococcus lactis* phage KSY1, originally isolated in Finland, is the only phage of the C3 type from Gram-positive bacteria that has been genome sequenced and analysed in more detail [12], [13]. Its head is much longer than the heads of the phages infecting enterobacteria, and its genome and genes show no similarity to the other C3 phages mentioned.

In this paper, we report the genome sequence and the morphological characteristics of a C3 morphotype phage, with special attention to the formation of the large elongated capsid. We have made comparisons of two of the structural genes of the C3 phages and their closest relatives within the *Podoviridae* family, and performed phylogenetic analyses. Mass-spectrometry based proteomics was employed to verify those proteins predicted by genomic analysis. Apart from ordinary TEM, we have also applied ultra-thin sectioning TEM and scanning electron microscopy.

## Materials and Methods

### Genome enrichment and purification

A 700 ml high titre stock were prepared and treated by DNaseI and RNaseA overnight in a 37°C shaking incubator. The DNase and RNase treated lysate was enriched using the polyethylene glycol (PEG) precipitation protocol [14] using PEG with an average molecular weight of 8000 (Cat. no. P5413, Sigma-Aldrich). The concentrated lysate was treated a second time by DNaseI and RNaseA for two hours. The virions were digested with Proteinase K treatment and heat treated, and the phage genomic DNA was precipitated in three consecutive steps adding 1/10 volume of 3M sodium acetate, pH 5.2, and 3 volumes of 100% ethanol. Phage genomes were extracted using Phase Lock Gel – 2 ml Heavy (Cat. no. 2302830, Sigma 5 Prime) with 25∶24∶1 phenol-chloroform-isoamyl alcohol (Cat. no. 77617, Sigma-Aldrich) as organic phase. The DNA was precipitated with ethanol following a standard protocol [14] using 96% ethanol and sodium acetate buffer at pH 5.2.

### Genome sequencing and annotation

After random shearing, the purified genomic phage DNA was sequenced utilizing the 454 pyrosequencing technology on a GS Junior system according to the recommendations and protocols of Roche/454 (Eurofins, Germany). The resulting reads (mean length 455) were assembled with the Newbler 2.6 software. Prediction of coding DNA sequences (CDSs) were performed with Glimmer 3.0 [15]. FindTerm (Softberry Inc.) was used for finding transcriptional terminators in the genome, and BPROM (Softberry Inc.) was used for finding promoters in the sequence [16]. All putative promoters, terminators and ribosomal binding sites were visually verified. The variation within the terminal repeats (TR) was analysed in Geneious 6.0 [17]. The genome has been submitted to NCBI and can be found under the GenBank accession number KM044272.

The prediction of secondary structure of the inferred amino acid sequences of the scaffolding proteins was done in psipred at http://bioinf.cs.ucl.ac.uk/psipred/, and corroborated with several other prediction programs (e.g. PSSfinder at http://www.softberry.com/or Jpred [18]).

The genome was scanned using tRNAscan-SE 1.21 [19] for tRNAs. BLASTX was run on each CDS, and BLASTP was applied on inferred amino acid sequences, in the search for similar proteins in the NCBI databases.

### Mass spectrometry-based proteomics

#### Sample preparation

The phage was concentrated by PEG precipitation, but not further purified by CsCl gradient centrifugation. Consequently, there might be contaminating bacterial proteins still in the sample, but phage proteins encoded by early or middle genes could also be present and possibly be detected. The contaminating bacterial proteins are discarded in the following data analysis (see below). The highly concentrated PEG precipitated phage samples were solubilized in lysis buffer containing 4% SDS (Sigma-Aldrich, St Louis, MO, USA), 1 mM dithiothreitol (DTT, Santa Cruz Biotechnology, Dallas, TX, USA) and 50 mM PBS heated at 95°C for 5 min followed by sonication for 1 min to shear DNA, and centrifuged at 14,000× g for 15 min. Protein concentration was determined using the Bradford method (Bio-Rad, Hercules, CA, USA). The supernatant was subjected to spin filter sample preparation (Centrifugation devices, NANOSEP; 10 kDa cut off, PALL, Port Washington, NY, USA) [20]. Briefly, soluble protein fraction was mixed with 8 M Urea buffer (8 M Urea, Sigma-Aldrich), 1 mM DTT in 25 mM HEPES, Sigma-Aldrich) and centrifuged at 14,000× g for 15 min. Following the reduction with 1 mM DTT and alkylation with 25 mM iodoacetamide (Sigma-Aldrich, St Louis, MO, USA) in 4 M Urea buffer (4 M Urea in 25 mM HEPES), the lysate was digested with trypsin (Sequencing grade modified trypsin, Promega, Madison, WI, USA) in 0.25 M Urea buffer (0.25 M Urea in 100 mM HEPES) at 37°C overnight, cleaned by a strata-X-C-cartridge (Polymeric SCX, Phenomenex, Torrance, CA, USA) and freeze dried in a SpeedVac system. Samples were stored at −80°C until analysis.

#### NanoLC-MS/MS analysis

Nano LC-MS/MS analysis was conducted using a hybrid LTQ-Orbitrap Velos mass spectrometer (Thermo Fischer Scientific, Waltham, MA, USA) equipped with an Agilent HPLC 1200 system (Agilent Technologies, Santa Clara, CA, USA). The injection volume was 1 µl and flow rate was 0.4 µl/min. Solvent A was 97% water, 3% acetonitrile (ACN), 0.1% formic acid (FA); and solvent B was 5% water, 95% ACN, 0.1% FA. The curved gradient went from 2% B up to 40% B in 240 min, followed by a steep increase to 91% B in 4 min. 1 µg of tryptic-digested sample was injected into a C18 guard desalting column (Zorbax 300SB-C18, 5 µm bead size, 5×0.3 mm i.d., Agilent Technologies, USA) prior to a 15 cm long C18 PicoFrit column (NTCC-360/100-5-153, 100 µm i.d., 5 µm bead size, Nikkyo Technos Co., Japan) installed on to the nano electrospray ionization (NSI) source. Precursors were isolated with a 2 *m/z* width and dynamic exclusion was used with 60 s duration. We enabled “preview mode” for FTMS master scans, which proceeded at 30000 resolution (profile mode). Data-dependent MS/MS (centroid mode) followed in two stages: first, the top 5 ions from the master scan were selected for collision induced dissociation (CID, at 35% energy) with detection in the ion trap (ITMS); and second, the same 5 ions underwent higher energy collision dissociation (HCD, at 45% energy) with detection in the orbitrap (FTMS). The entire duty cycle lasted ∼3.5 s.

#### Data Analysis

The raw files from the Orbitrap analysis were searched by SEQUEST under the software platform Proteome Discoverer 1.4.0.288 (DB version: 79, Thermo Fischer Scientific) against the predicted proteins inferred from the phage vB_EcoP_SU10 genomic analysis using a 99% confidence cut off limit. A precursor mass tolerance of 10 ppm and product mass tolerances of 0.02 Da for HCD-FTMS and 0.8 Da for CID-ITMS were used. Further settings used were trypsin with 1 missed cleavage; carbamidomethylation on cysteine as a fixed modification; oxidation of methionine and carbamidomethylation on N-terminal as variable modifications.

### Phylogenetic analyses

Treatment of sequences and the phylogenetic analyses were carried out in MEGA6 [21]. The inferred amino acid sequences from CDS8 (scaffolding protein) and CDS9 (major head protein) were aligned by Clustal Omega [22]. A maximum parsimony (MP) tree was generated for both sequences and compared. Since both genes showed the same phylogeny with the exception of the position of KSP100, the subsequent analyses were done on the concatenated amino acid sequence. KSP100 was closer to the root when the head protein was analysed than when the scaffold protein was analysed but the node had poor support in the latter case. The concatenated amino acid sequence was subjected to a test of character evolution resulting in a best fit by the Whelan and Goldman (WAG) model [23]. The following analyses were done using the maximum likelihood method with the shortest MP tree as initial tree in heuristic searches under the WAG model. Confidence in the nodes of the tree with the highest likelihood was evaluated in a bootstrap analysis with 500 replicates. Different rates of character change in the tree were tested with Tajima's relative rates test, and the molecular clock test using the maximum likelihood method, both implemented in MEGA6. The final tree including divergence times of the two concatenated structural genes was also calculated in MEGA6.

### Electron microscopy

#### Transmission electron microscopy

Phage particles of a PEG precipitated lysate were deposited on carbon-coated grids, negatively stained with 1% uranyl acetate, and observed in a Tecnai G2 transmission electron microscope at 80 kV.

#### Ultra-thin section electron microscopy

Specimens were preliminary fixed in 2.5% glutaraldehyde in 0.1M phosphate buffer, pH 7.4 and stored at +4°C. Fixed cells were rinsed in 0.15 M sodium cacodylate buffer and centrifuged to pellet the cells. Post fixation of cells was done in 2% osmium tetroxide in 0.1M phosphate buffer, pH 7.4 at +4°C for 2 hours, dehydrated in ethanol followed by acetone, and embedded in LX-112 (Ladd, Burlington, Vermont, USA). Embedded cells were sectioned (ultrathin sections approximately 50–60 nm) using a Leica EM UC6 (Leica, Wien, Austria). Sections were contrasted with uranyl acetate followed by lead citrate and examined in a Tecnai 12 Spirit Bio TWIN transmission electron microscope (FEI Company, Eindhoven, The Netherlands) at 100 kV. Digital images were taken by using a Veleta camera (Olympus Soft Imaging Solutions, GmbH, Münster, Germany).

#### Scanning electron microscopy

Fixation was done by immersing of specimens in 2.5% glutaraldehyde in 0.1M phosphate buffer, pH 7.4, followed by rinsing in 0.15 M sodium cacodylate buffer. Fixed specimens were attached to a RC58 filter (Merck Euro lab, Darmstadt, Germany), briefly rinsed in distilled water, and stepwise dehydrated in 70% ethanol for 10 min, 95% ethanol for 10 min, absolute ethanol for 15 min, and finally in acetone for 10 min. The dehydration was done at +4°C. Specimens were then dried using a critical point dryer (Balzer, CPD 010, Lichtenstein) cooled with dry ice. Dried specimens were mounted on aluminium stubs and coated with Carbon (Bal-Tec MED 010, Leica Microsystems, Liechtenstein). The specimens were analysed in an Ultra 55 field emission scanning electron microscope (Zeiss, Oberkochen, Germany) at 3 kV.

## Results and Discussion

### Genome characterization and annotation

*De novo* assembly of the resulting reads from the SU10 genome sequencing resulted in a single contig of 77,134 base pairs (bp), with a very high average sequencing coverage of 978. Many features of the SU10 genome showed a high resemblance to the genome of phiEco32. The G+C content was for instance 42.1%, which was very close to the 42.3% G+C content of phiEco32. With the aid of the sequences of the terminal repeat (TR) of phiEco32, the TR region ends of the genome were identified and shown to be of the same size, 193 bp, and about 94% identical to the TR's of phiEco32. Since the TR's at both ends of the genome were identical, they were aligned in the resulting contig file. Hence, the actual overall genome size was 77,327 bp, slightly shorter than the phiEco32 genome. The two direct TR's were sieved out from the raw sequencing reads, by using the bases immediate before and after the repeats as probes, and examined. The reads all showed a sharp drop-off after the last nucleotide in each TR, which indicated a genome with blunt ends. The result also showed that they were not all identical, and in contrast to phiEco32 which only showed minor variation in the left TR and a constant right TR [24], there were variation at both ends. There was another direct repeat within the TR's, CCCTTTTTTA – 10 nt – CCCTTTTTTA, located 21 nt from the end of each TR, which varied between reads. The variation was always a deletion of one of the 6 thymines. There were never deletions in both internal repeats at the same time, and most frequently in the first repeat of the left TR. The first direct repeat in the left TR was thus CCCTTTTTA in 25% of its repeats (n = 536), but only 10% in the right ending TR repeat (n = 170). Curiously, there is sometimes a deletion in the second direct repeat in the left TR, but never in the second direct repeat in the right TR. These differences are remarkable, and since quite a number of reads were examined, we think that the differences reflect unspecific cleaving of the concatemers. It has been suggested that these phages have a T7 type of genome replication, where new DNA is synthesised almost to the 3′ end, forming an overhang that joins with other incomplete duplexes into concatemers. Before DNA packaging, these concatemers need to be cleaved but the cleavage is complicated and take place in several steps since the repeats also need to be duplicated [25]. There are models that hypothesise how this is achieved, involving strand displacement synthesis or primase initiated synthesis. A hairpin structure in the DNA containing the TR is found during T7 infection, which supports the latter [26], but both models involve nicking at different stages where errors may be generated. The variation could also arise as a consequence of polymerase stuttering during the following synthesis, but the 5-thymine half repeat is found in both the left and the right TR and we believe that the variation found would be more or less equal in both TR's if it was the result of stuttering. It was of course impossible to know if the 5- thymine half repeat is present in both TR's in the same genome due to the sequencing of a multitude of genomes, but this could possibly indicate that there are two variants of the genome present in nature. In a third model, it has been shown that the T7 RNA polymerase (RNAP) has an important role in the recruitment of the prohead and the terminase complex [27]. However, the phiEco32 like phages lack a RNAP of their own, which point to that these phages may have a completely different and unknown mechanism for generation of complete genomes and genome packaging.

A total of 125 coding DNA sequences (CDSs) encoding polypeptides of at least 38 amino acids were found and 85 of them had identifiable ribosomal binding sites. In addition, 4 Rho-independent bacterial terminators were identified (Table 1). In general, the SU10 genome structure shows high similarity to that of phages phiEco32, NJ01, KBNP135, and KBNP1711 (The genome of KBNP1711 was published after the initial submission of this manuscript and is not included in the following analyses). However, there are some differences in gene content (S1 Table). According to BLASTP searches, SU10 have 111 CDSs that show similarity to those of phage phiEco32, 89 with NJ01, 96 with KBNP135, 40 with 7–11 and 28 with GAP52.

**Table 1:** General features of the phage SU10 genome.

Genome size	77,327	Ribosomal slippages	1
G+C content	42.10%	Similarity^1^ to phiEco32	111 CDSs
CDSs	125	Similarity to KBNP135	96 CDSs
Predicted proteins^2^	38	Similarity to NJ01	89 CDSs
RBS	85	Similarity to 7–11	40 CDSs
Promoters	13	Similarity to GAP52	28 CDSs
Rho-independent terminators	4	Early genes	69–125, 1–3
		Middle genes	25–68
		Late genes	4–24

1 Over 40% similarity between the encoded gene products.

2 Proteins with a known function predicted by genomic analysis (BLASTP).

The SU10 proteins gp55, gp63 and gp123, were not present in other C3 morphotype phages but were similar to genes in unrelated phages or bacteria, a fact that demonstrates the mosaic pattern often encountered in phage genomes. Gp63 was shown to be similar to a hypothetical protein in *Coleofasciculus chthonoplastes* PCC7420, and gp55 similar to a hypothetical protein in the *Cronobacter* phage vB_CsaM_GAP31.

The SU10 genome was shown to be most similar to the genomes of other *E. coli* phages with C3 morphology. Whole genome comparison studies using CoreGenes3 [28] showed that it shared 85.2% homologous genes with phage phiEco32 and 80.7% with NJ01. The SU10 genome was slightly less similar to the two *E. coli* phages KBNP1711 (72%) and KBN135 (69%), and even less to 7–11 (29%) and GAP52 (29%). SU10 also showed the same overall genome architecture as phage phiEco32, and the genome can be divided into the same early, middle and late genes [6], [24]. The similarity to other phages with C3 morphology not mentioned above was limited to a few structural genes.

#### Early genes

**Figure 2:**
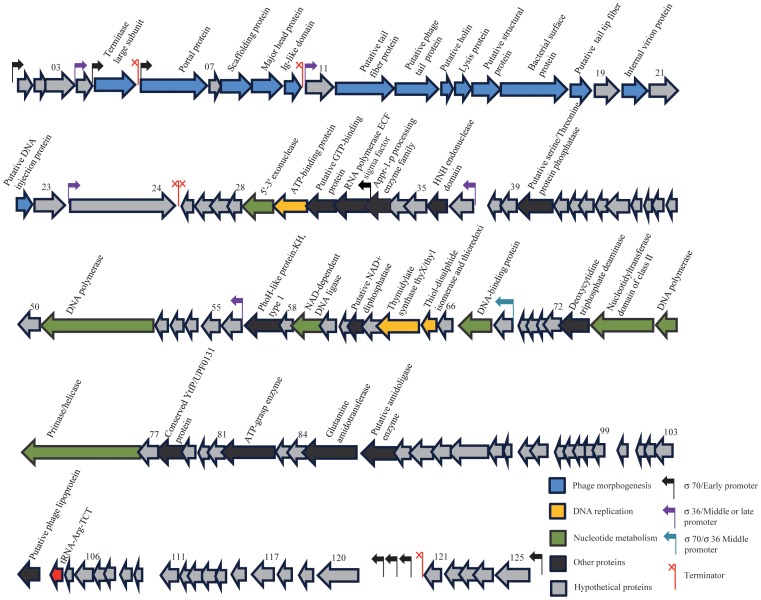
The genome of phage SU10. The predicted CDSs are indicated by arrows, and the directions of arrows indicate direction of transcription. 28% of the predicted proteins were identified in our mass-spectrometry based proteomics. CDSs predicted to encoding structural proteins are indicated in blue, hypothetical proteins in grey, DNA replication and recombination proteins in green. Genes predicted to encode enzymes involved in nucleotide metabolism are indicated in yellow, black is showing other proteins.

CDSs 69–125 on the genetic map (Fig. 2) constitutes the early genes, and like phage phiEco32 there is one promoter upstream of CDS125 with a putative terminator downstream of CDS121, and three promoters in the spacer region between genes 120 and 121 that should be recognized by the RNA polymerase holoenzyme containing the σ^70^ factor. In addition, CDSs 1–10 located at the other end of the genome, are preceded by hypothetical σ^70^ promoters (S2 Table), and are therefore also expected to be expressed early after infection. The region contains many short CDSs but all are preceded by potential ribosomal binding sites and might thus be expressed. The majority of the hypothetical gene products show an identity above 90% to phiEco32, and in most cases with a high identity to CDSs found in phages NJ01 and KBNP135 (S1 Table). Products involved in DNA replication, repair, recombination, transcription, metabolism and cell lysis have a very high similarity also to phages 7–11 and GAP52. Only two CDSs are not found in any of the other C3 phage types, namely CDS123 which has a weak similarity to a potential isoleucyl-tRNA synthetase with 31% identity over 54 residues in *Arthrobacter sp FB24*, and CDS77 that is unique. The early genes do not end with a detectable termination signal, indicating that transcription may continue into the middle genes.

#### Middle genes

By homology to phiEco32, CDSs 25–68 should constitute the middle genes. A σ^70^ promoter is located upstream of CDS68, with a good -35 and -10 region, and two more with poor -35 regions, one between CDSs 56 and 57 and another between CDS37 and 38. The presumptive sigma factor of SU10 is encoded by CDS32, which has its own promoter showing the *E. coli* consensus -35 and -10 regions but the promoter half-sites are spaced by 18 nucleotides which might lead to reduced transcription. The inferred protein of CDS32 shows 100% identity to the sigma factor of phiEco32 (gp36) indicating that these two phages have similar late promoters. The three middle promoters and the three late promoters of phiEco32 are recognized by the sigma-36 factor and a common motif tAATGTATAtA has been identified 6–8 nt upstream of the transcriptional start points [24]. The same motif can be found in the three middle promoters of the SU10 phage, indicating that the middle genes can be expressed by both the σ^70^ holoenzyme and by the phage RNA polymerase with the gp32 sigma factor. Phage phiEco32 encodes a small protein; (gp 73) that binds to and inhibits σ^70^ polymerase, but it binds to and activates some, but not all of the middle and late sigma-32 promoters [24]. This protein is conserved with 99 and 95% identity in SU10 (gp73) and KBNP135 respectively, and to about 50% in phages 7–11 and GAP52, indicating a similar regulatory role in these phages. A putative Rho-independent transcriptional terminator is located downstream of CDS25 (S2 Table). The middle genes of SU10 encodes 44 possible proteins, and 37 of them are present in phiEco32 with over 90% identity, except CDS45, 53, and 54 that are 86, 89 and 66% identical respectively. Other CDSs are present in one or several of the related C3 phages except CDS36, CDS55, and CDS63 that are unique. CDSs that are present in all five sequenced C3 phages are CDS29, CDS37, CDS41, CDS51, CDS56, CDS65 (thioredoxin), CDS67 (DNA binding protein, contains a DPS (DNA Protecting protein under Starved conditions) domain), and CDS68, where the numbers corresponds to those in the SU10 genome.

#### Late genes

The late genes correspond to CDS4 to 24. There are three promoters that contain the consensus motif tAATGTAt, one located between CDSs 23 and 24, another between CDSs 10 and 11, and a third between CDSs 3 and 4. Thus, CDSs 4–10 might be expressed both by the host σ^70^ polymerase and by the polymerase with the SU10 sigma factor. Possible terminators are located in the spacers between CDSs 10 and 11, as well as between CDSs 5 and 6 (S2 Table). The SU10 structural genes show over 90% identity to those of phiEco32 with the exception of the tail protein (SU10, gp13), and the tail fibre protein (SU10, gp12) that show 67% and 82% respectively. This may reflect a faster evolution of the receptor recognition domains of the phages. All the structural proteins are well conserved among the related phages, SU10, phiECO32, NJ01 and KPNB135. Furthermore, the capsid proteins are conserved among all six C3 phages, while the tail proteins seem to differ. Also the holin and lysozyme genes are well conserved in phages SU10, phiEco32, NJ01 and KPNB135, but they are not found in 7–11 and GAP52. A potential ribosomal slippage (GGGAAAG) was identified in the presumed major head protein (SU10 CDS9) between nucleotide 8,390 and 8,396 which would lead to a fusion of the major head protein with the Ig-like domain of CDS10 as in phage phiEco32 [6]. The ribosomal slippage site is part of a sequence that can form a stem-loop structure (CTGCTGCGGGAAAGGCGGCGG) as identified by Mfold [29]. The stem-loop structure may interfere with the ribosome and thus regulate the production of the fusion protein.

### Proteomics

To identify the SU10 predicted proteins by our genomic analysis, the phage was amplified, concentrated and analysed using mass spectrometry. 34 proteins were identified with a False Discovery Ratio (FDR) of 1% (Table 2), and 22 out of these were identified as having a known function. Seven of the identified proteins can be categorized as structural proteins, proteins involved in the morphogenesis of the phage (gp6, gp8, gp9, gp13, gp12, gp16 and gp18). The major head protein (gp9) comes in two alternative forms of 38.5 and 55.4 kDa, respectively, supporting a ribosomal slippage fusing of gp9 and gp10. A portal protein with molecular mass of 85.0 kDa (Table 2) encoded by CDS6 had the highest coverage (78.5%) and highest number of peptides (50). The internal virion protein (gp20) and the DNA injection protein (gp22) were identified by sequence coverage of 39.6% and 49.1% respectively, and were also classified as phage morphogenesis proteins. The terminase large subunit (58.1 kDa), involved in DNA packaging of the phage, was identified by 2.14% sequence coverage and a single peptide. The products of CDS51 (DNA polymerase, 69.1 kDa) was identified by 6.7% sequence coverage and 2 peptides and CDS67 (DNA-binding protein, contains a DPS domain) 21.2 kDa with 35.8% sequence coverage and 6 peptides. Thiol-disulphide isomerase/thioredoxin (gp 65) involved in DNA replication was identified with 52.8% sequence coverage and 4 peptides. The observed molecular mass of identified proteins is in general very close to the predicted mass for the proteins. The mass-spectrometry based proteomics identified 22 of the 38 proteins, predicted in our genomic analysis (Table 1), with a known function. In total, 34 out of 125 (28%) predicted proteins were identified by mass-spectrometry proteomics, most of them encoded by identified structural genes.

**Table 2:** Proteins identified by hybrid LTQ-Orbitrap Velos mass spectrometer.

CDS	Exp. MW (kDa)^1^	Id. MW (kDa)^2^	Putative function	Sequence coverage (%)	Peptides (No)
**Late gene products**					
9	38.6	38.5	Major head protein	78.1	23
9+10	55.4	55.4	Major head protein (alternative)	50.29	22
8	41.2	41.2	Scaffolding protein	54.5	21
6	85	84.9	Portal protein	78.5	50
13	78.1	78.1	Phage tail protein	40.1	23
12	83.5	83.4	Tail fiber protein	64.5	34
18	35.3	35.3	Tail tip fiber protein	75.2	12
16	29.2	29.2	Structural protein, base wedge/pin domain	73.8	15
17	110	110	Bacterial surface protein	51	37
22	33.3	33.3	DNA injection protein	49.1	5
20	27.5	27.5	Internal virion protein	39.6	9
24	163	163	Hypothetical protein	73.2	116
23	60	59.9	Hypothetical protein	52.1	32
11	28.8	28.8	Conserved protein, unknown function	54.4	17
21	35.9	35.9	Hypothetical protein	53.4	11
3	24.1	24.1	Hypothetical protein	30	3
5	58.2	58.1	Terminase large subunit	2.14	1
**Middle gene products**					
51	69.2	69.1	DNA polymerase	6.68	2
67	21.2	21.2	DNA-binding protein	35.8	6
65	10.1	10.1	Thiol-disulphide isomerase and thioredoxin	52.8	4
57	27.6	27.6	PhoH-like protein:KH, type 1	30.6	3
32	25.8	25.8	RNA polymerase ECF sigma factor	8.41	1
31	14.6	14.6	GTP-binding protein	15.4	1
56	10.8	10.8	Hypothetical protein	18.3	2
27	15.3	15.3	Conserved hypothetical protein	18.8	1
68	12.2	12.2	Conserved hypothetical protein	22.9	2
55	11.6	11.6	Hypothetical protein	18.6	1
47	6.32	6.31	Hypothetical protein	70.9	2
**Early gene products**					
82	44.5	44.4	ATP-graze enzyme	23.8	6
85	63.8	63.7	Glutamine amidotransferase	23.7	9
78	15.9	15.8	Conserved YtfP/UPF0131 protein	33.8	2
86	23.3	23.2	Hypothetical protein	10.2	1
74	50.9	50.9	Nucleotidyltransferase domain of class II CCA-adding enzyme translation	7.24	1
91	13.9	13.9	Hypothetical protein	36.7	3

1 Expected molecular weight for identified proteins.

2 Identified molecular weight by mass-spectrometry based proteomics.

### Morphological analyses

**Figure 3:**
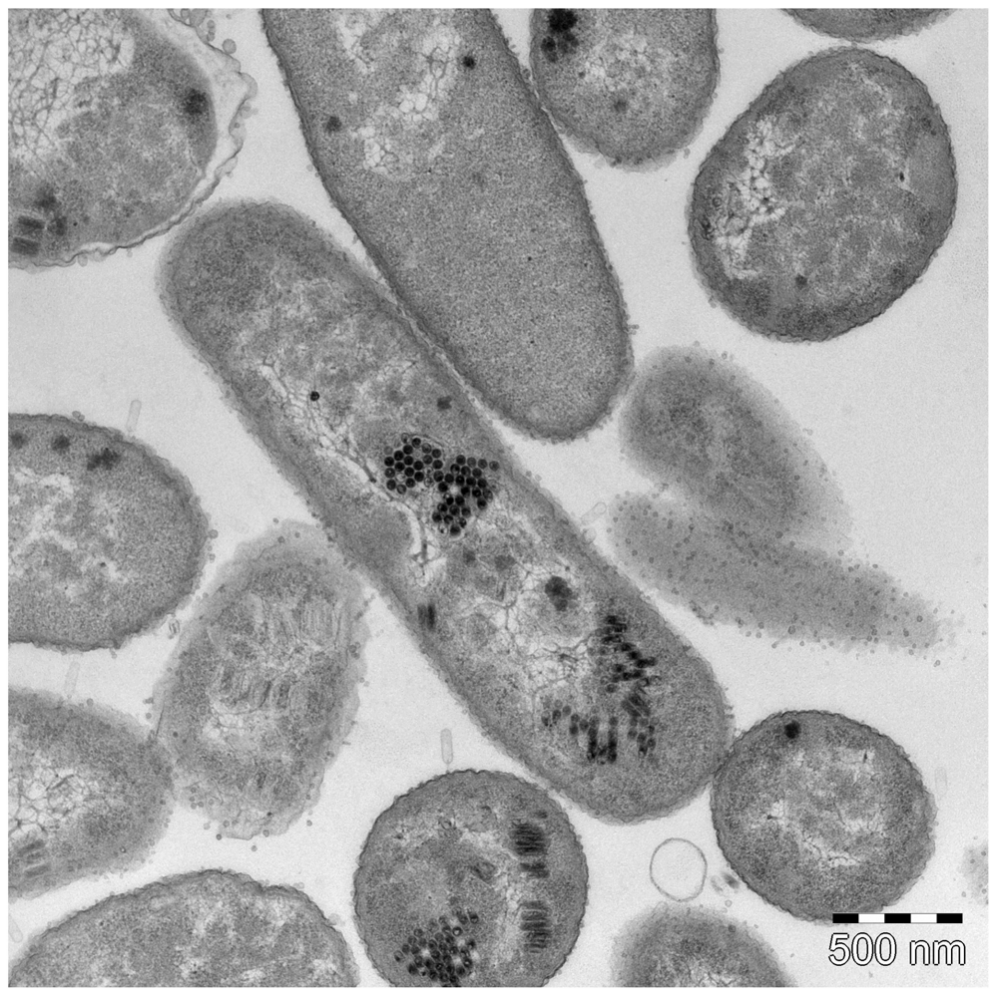
Ultra-thin section electron microscopy SU10. Phage empty capsids are attached to the bacterial surface receptors and their genomes are injected to the host. Phage progenies heads are standing side by side in the bacterial cytoplasm and forming a honeycomb shape structure. Some of the heads are filled by genomes and some are in the stage of genome packaging.

TEM images show that the genomic similarity of SU10 to phage phiEco32 is also reflected in its morphology, the two phages are very similar (Fig. 1). The capsid of SU10 seems to be shorter and wider than phiEco32, 137×57 as compared to 145×44 nm, and the tail to be a little longer, 19 nm. The capsid and tail sizes vary between the closely related phages SU10, NJ01, and phiEco32, but their tail/head ratio is the same, 0.1. An explanation to the size difference can be that these phages' capsids are often observed in a flattened shape, and not appearing as rod shaped. To get a better picture, and possibly avoid artefacts caused by the TEM preparation, we prepared SEM images of the phages during infection of the cells (Fig. 3). The images showed empty capsids, with their tails attached to the cell surface, perfectly rod shaped with faceted ends.

**Figure 4:**
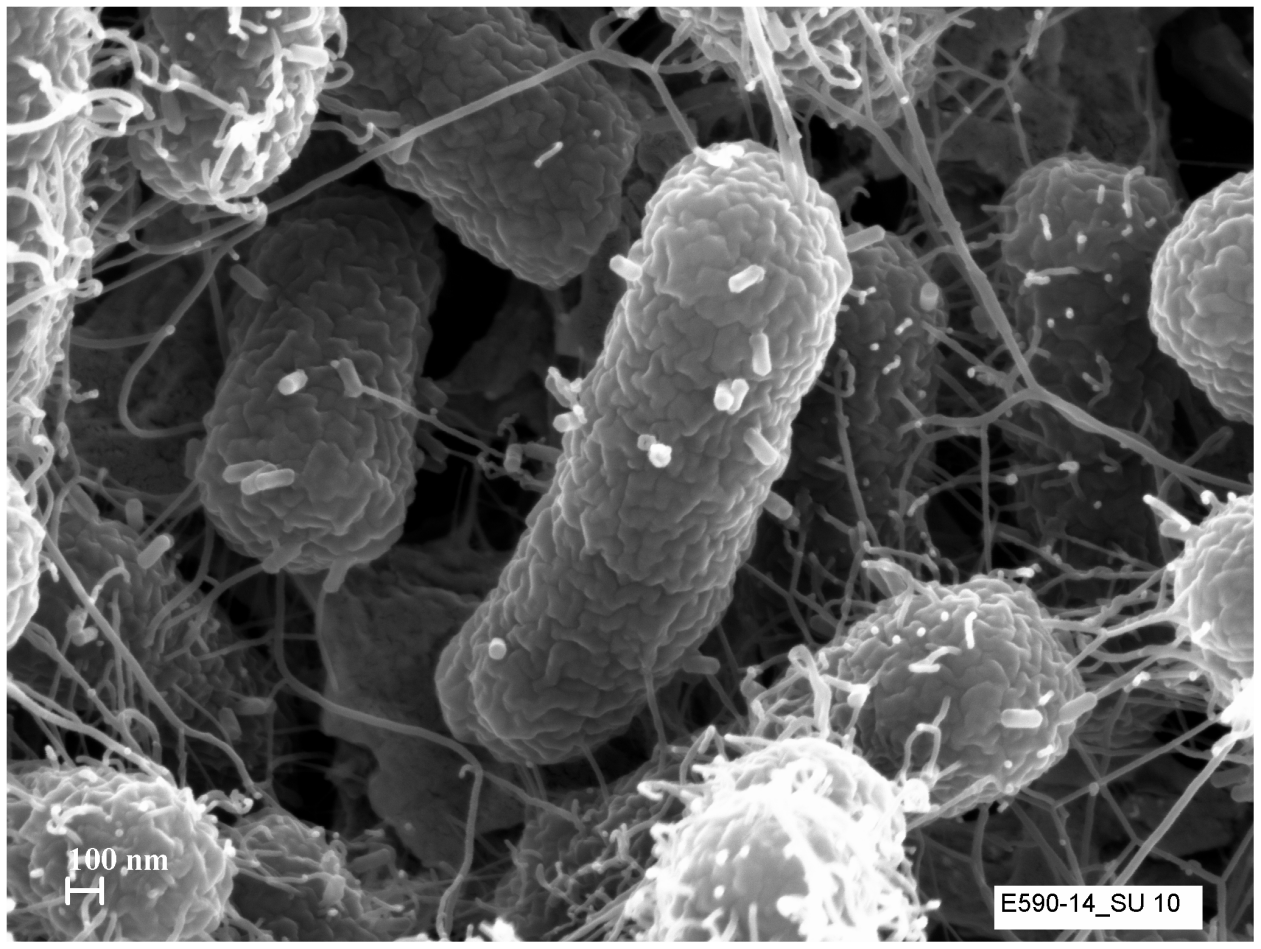
Scanning electron microscopy image of SU10 five minutes after adding them to the bacterial culture. Empty phage particles can be seen adsorbed onto the surface of the host cell.

The ultra-thin sectioning images revealed that the SU10 phages were arranged into packages inside the cells during DNA packaging (Fig. 4). Contrary to other phages at this stage in the life cycle, the capsids were never randomly distributed. The packages were often oriented perpendicular to the length axis of the cell, but when not, it was possible to observe the different stages of the DNA packaging. It appears that the capsids are already in their final shape and size before DNA packaging starts, which implies that the DNA itself cannot function as a template or play a role in the formation of the longer capsid. Furthermore, a comparison between the size of C3 morphotype genomes and their corresponding capsid size showed a lack of correlation which also suggests that the length of the DNA molecule is not affecting the elongation of the capsid. It is likely that the packages are formed where the concatemers of the genomes are processed into single units, and that each package is the result of one infecting phage. This would also imply that the transcription of the late genes and assembly of the phage particle is linked to this process.

It was also possible to observe infecting phages in the ultra-thin sectioning images. Surprisingly, these phages seem to have longer tails than observed in the ordinary TEM images, which suggests that the tails change their conformation in connection with, or after, the injection of the genome. The tails at this stage were measured to be 41 nm, more than twice the size of tails observed on free phage particles in ordinary TEM imaging (19 nm), and three times as long as the phiEco32 tail.

The package arrangement of capsids may also suggest an alternate role for the scaffolding protein during the assembly. It is conceivable that the scaffolding protein builds a honeycomb-like structure at the same time as the major head protein monomers are incorporated. We made comparisons of the predicted secondary structure of the scaffolding proteins of C1 and C3 phages. The C1 phages have an icosahedral capsid and the secondary structure of the scaffolding proteins is always an α-helix in the N-terminal followed by a β-sheet and another very long α-helix through to the C-terminal end. The C3 phages have the same long C-terminal α-helix, but the secondary structure in their N-terminal end varies from phage to phage. For instance, the C3 phage GAP52 lacks the N-terminal α-helix completely, and SU10 and phiEco32 both have two 4–5 residues short α-helices in their N-terminals (S1 Fig.). Thus, the scaffolding proteins of the C3 morphotype phages are different from one another and more heterogeneous at the N-terminal end than what is common among C1 morphotype phages, but it is impossible to establish that such differences can cause an elongation of the capsid.

### Phylogenetic analyses

**Figure 5:**
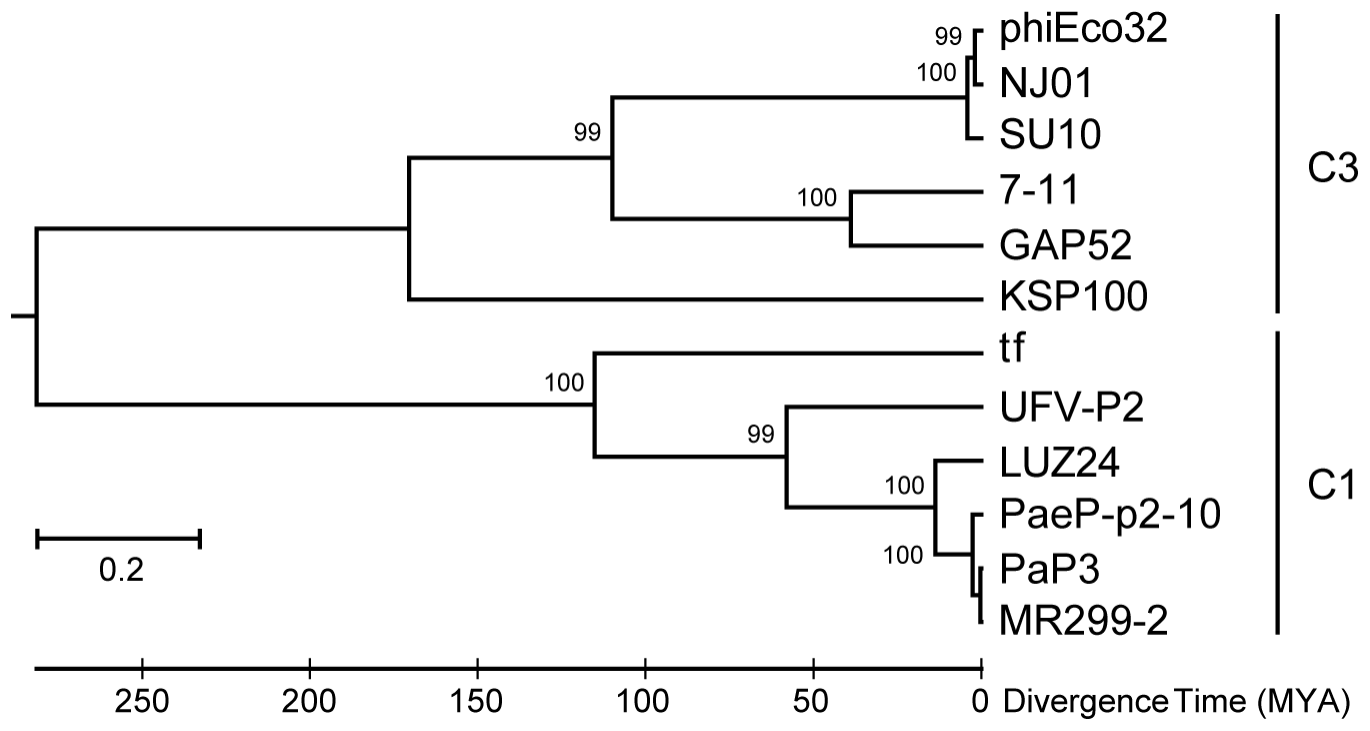
Phylogenetic tree showing the relationship of the two structural proteins, the scaffold protein and the head protein, within and between the two morphotypes. Phages SU10, NJ01, PhiEco32, GAP52, 7–11, and KSP100 belong to the C3 morphotypes (elongated capsid), and phages tf, UVF-P2, LUZ24, PaeP-p2-10, MR299-2, and PaP3 to the C1 morphotype (icosahedral capsid). The tree was inferred by using the Maximum Likelihood method based on the Whelan and Goldman model [23]. The tree with the highest log likelihood (−9785.9307) is shown. A maximum parsimony (MP) tree was used as an initial tree in the heuristic search. The tree is drawn to scale, with branch lengths measured in the number of substitutions per site. The analyses were conducted in MEGA6 [21].

The scaffolding protein gene and the major head protein gene were the only genes of the C3 phages where the corresponding genes were found among C1 phages. All of these were found in phages of *Pseudomonas*. The phylogenies of the scaffolding protein and the major head protein residues were similar enough to be merged into a common phylogeny since the phylogeny of these genes showed the same two groups, one group with the phages with C3 morphology, and the other with the more common C1 or T7-like morphology (Fig. 5). Since the scaffolding protein gene and the major head protein gene of the C3 phages follow the same evolution, it can be assumed that the scaffolding gene is not a recent addition to the genome that has transformed the function and assembly of the head protein resulting in an elongated capsid. Both trees had the same topology with the exception of the KSP100 scaffolding protein which was more closely related to the GAP52 – 7–11 cluster in the scaffolding protein tree as compared with the head protein tree. The sequence differences were more pronounced in the scaffold sequences than in the head sequences. The resulting single tree thus showed the same phylogeny as the head tree with good support for all nodes (Fig. 5). Midpoint rooting of the tree, as well as a distance tree of the alignment, displayed two major clusters; one containing all the C3 phages, and the other all phages with an icosahedral C1 morphotype. Phage tf (C1) and phage KSP100 (C3) were closest to the root both representing more plesiomorphic states of the characters. Phylogenetic analysis of phage genes, or inferred amino acid sequences, is problematic because of horizontal transfer of genes and recombination between phage genomes, leading to the mosaic genomes that are typical for phages. Rooting of trees becomes equally difficult since it is impossible to find a suitable outgroup. This is, to our knowledge, the first time that rooting of phage genes has been possible. This was possible because the genes belong to phages from two different morphological groups, although they show similarities at the gene level, and the clustering of all the C3 genes point at that they have a common origin. In addition, the C1 and C3 genes are from phages infecting bacteria of two different γ-proteobacteria families, Pseudomonadaceae and Enterobacteriaceae, which must have separated unknown millions of years ago. The capsid genes of C3 phages of bacteria belonging to Enterobacteriaceae are closely related only to capsid genes from phages with C1 morphology infecting bacteria from Pseudomonadaceae. There are no similar capsid genes among other phages, e.g. C1 morphotype phages, infecting bacteria belonging to Enterobacteriaceae. The time since divergence of *Escherichia* and *Salmonella* has however been estimated to be 100 million years, based on the age of mammals as they are the hosts for *Escherichia coli* [30]. Assuming the same rate of evolutionary change throughout the phylogeny of the C1 and C3 genes as between *Escherichia* and *Salmonella*, represented by SU10 and 7–11 in the tree, the time since divergence between the two morphotype genes becomes roughly 280 million years. The rate of change may however not be the same in all branches, since phages have very different life histories. Tajima's relative rate tests [21], [31], using three different combinations of branches and outgroup, resulted in one significant difference in rates between lineages. The other test, using the maximum likelihood method, also rejected the hypothesis of equal rates. Although two tests out of four show that equal rates may apply, the molecular clock is probably not the same in all branches and the calculated time since divergence between the C1 and C3 structural genes is thus a somewhat rough estimate. The time since divergence is nevertheless close to the time when tetrapods colonizing land first appeared. This might indicate that *Pseudomonas*-like bacteria where the first to colonize the intestines of these early tetrapods, and that these are ancestors to today's enterobacteria.

The *Lactococcus* phage KSY1 was isolated from traditionally fermented milk and does not show any relationship to the other C3 phages [12]. In an effort to investigate the phylogenetic relationship of KSY1, we tried to find its scaffolding gene by analysing the presumed secondary structure for putative proteins in the genome. We could however not find any protein with the typical long α-helix motif. The only gene apart from the major head protein gene that can be found in many other phages was the collar protein gene. Phylogenies of these two genes were very similar and indicate that the closest relatives of KSY1 are phages isolated on *Weissella* and *Streptococcus*. Although phage KSY1 is a singleton (there is a closely related phage, KSY2, but it has not been genome sequenced [32]), the most rational explanation to its C3 morphology in comparison to the other C3 phages is that it is a case of convergent evolution. A large genome is more costly to reproduce and the limited resources available from the host cell restricts the number of progeny phages, but at the same time, a large genome can contain more non-essential genes that only are needed to overcome bacterial phage resistance systems. The C3 phages have been shown to have wide host ranges [6]. We hypothesize that this is a trade off against their larger genomes, and that one explanation to their scarcity might be that they are more common in environments were bacteria are fewer but more varied, which would demand a more versatile genome.

## Concluding Remarks

The genome of SU10 is very closely related to phage phiEco32, and other enterobacterial phages with the C3 morphotype. These phages have an unknown mechanism for replicating and packaging their genomes, and for assembly of the capsid, which we think is quite distantly related from other *Podoviridae* phages, e.g. T7. The size of the capsid varies between C3 phages, but is specific for each one of them, and a genetic factor must be present that determines their lengths. The structure of the scaffolding proteins of C3 phages vary, and could explain the dissimilar elongation, but many genes in the genomes are unknown and there are other hypotheses as well. For instance, there could be another unknown protein that functions as a “tape measure” for capsid length, or another helper scaffolding protein that directs the assembly of the major head proteins into an elongated procapsid. The ultra-thin sectioning images reveal distinct packages of fully formed capsids being packaged with DNA. It is not far-fetched that the somewhat unspecific cleavage of the concatemeric DNA takes place when another particle in the honeycomb-like structure is formed, and possible also to the transcription of specific structural genes. Another feature that points to a rather distant relationship with other *Podoviridae* phages is the length and conformational change of the tail.

The phylogenetic analyses show clearly that the structural genes of *Podoviridae* C3 phages have coeveolved and that both diverged from the C1 structural genes a very long time ago. *Podoviridae* C3 phages are thus not structurally modified C1 phages, or the result of some kind of phase shifting. The division of the proteins in the phylogenetic analysis coincides with the two morphotype groups. Structural genes have been shown to coevolve and to be a quite stable module of phage genomes. They often form the core of what is used as the foundation for phage taxonomy. That, and the division into the two groups, makes it possible to root the tree of the structural proteins, and with some confidence also to estimate the time since the two groups parted. The result, even if molecular clocks and dating of evolutionary events of phages are prone for errors, is in agreement with the evolution of the bacteria that colonizes the gut of terrestrial tetrapods.

Having an elongated capsid could be an evolutionary advantage although it requires more energy for propagation. The elongated C3 morphotype has most likely originated at least twice, within phages infecting enterobacteria as well as lactococci, and is a nice example of convergent evolution.

## Supporting Information

S1 Fig **Protein secondary structure predictions of the N-terminal end of scaffolding proteins inferred from C3 morphotype phage genomes (1–5) and phages with C1 morphotype genomes (6–9).**
(TIF)

S1 Table **Proteins inferred from the SU10 genome sequence and results of BLASTP searches for similar proteins in other phages with C3 morphotype.**
(DOCX)

S2 Table **Early and middle transcription promoter and terminator sequences and their location in the genome of SU10.**
(DOCX)
