# Correction: Genomic, Proteomic, Morphological, and Phylogenetic Analyses of vB_EcoP_SU10, a *Podoviridae* Phage with C3 Morphology

**DOI:** 10.1371/journal.pone.0124730

**Published:** 2015-04-07

**Authors:** The PLOS ONE Staff

The first author’s name is cited incorrectly. The correct citation is: Khan Mirzaei M, Eriksson H, Kasuga K, Haggård-Ljungquist E, Nilsson AS (2014) Genomic, Proteomic, Morphological, and Phylogenetic Analyses of vB_EcoP_SU10, a *Podoviridae* Phage with C3 Morphology. PLoS ONE 9(12): e116294. doi:10.1371/journal.pone.0116294. The publisher apologizes for the error.
