# Supplementary figures and images for: Genomic, Proteomic, Morphological, and Phylogenetic Analyses of vB_EcoP_SU10, a Podoviridae Phage with C3 Morphology
Source: PLoS One. 2014 Dec 31;9(12):e116294. doi: 10.1371/journal.pone.0116294 (PMC4281155; doi:10.1371/journal.pone.0116294)

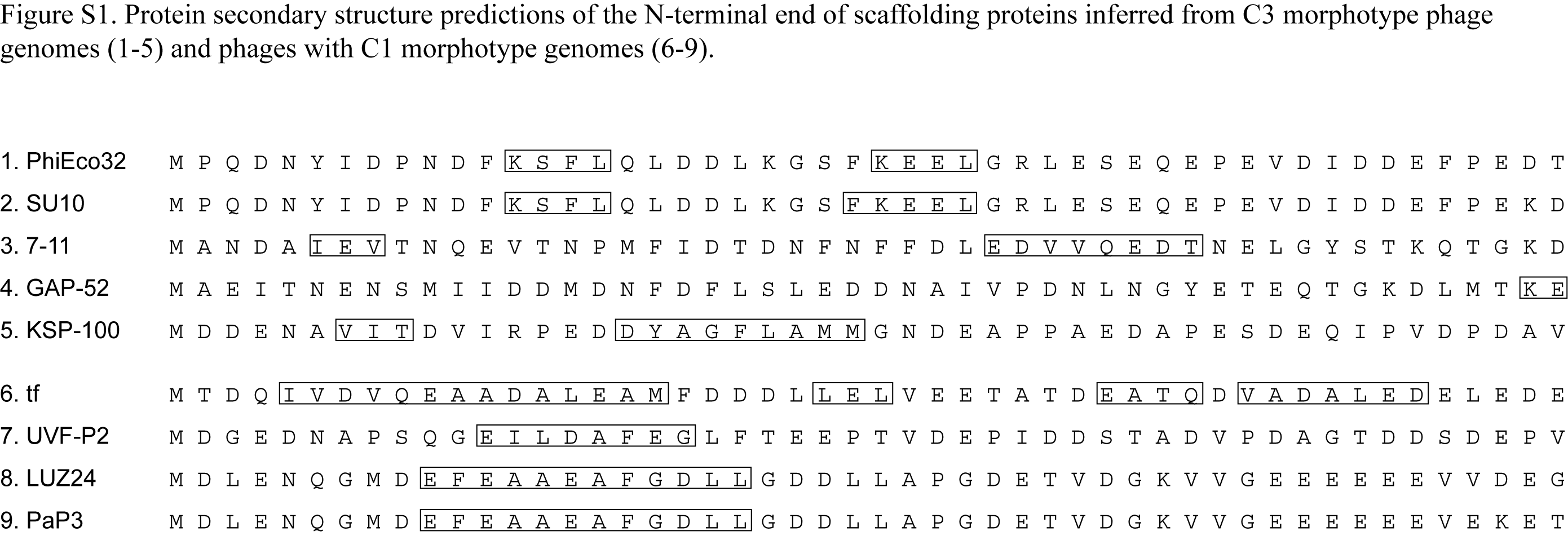

Supplement: S1 Fig — Protein secondary structure predictions of the N-terminal end of scaffolding proteins inferred from C3 morphotype phage genomes (1–5) and phages with C1 morphotype genomes (6–9). (TIF) [file pone.0116294.s001.tif]
