# Supplementary material for: Genomic, Proteomic, Morphological, and Phylogenetic Analyses of vB_EcoP_SU10, a Podoviridae Phage with C3 Morphology
Source: PLoS One. 2014 Dec 31;9(12):e116294. doi: 10.1371/journal.pone.0116294 (PMC4281155; doi:10.1371/journal.pone.0116294)
Supplement: S1 Table — Proteins inferred from the SU10 genome sequence and results of BLASTP searches for similar proteins in other phages with C3 morphotype. (DOCX) [file pone.0116294.s002.docx]

**Table S1**. Proteins inferred from the SU10 genome sequence and results of BLASTP searches for similar proteins in other phages with C3 morphotype.

|  |  |  | Identity to inferred proteins from related C3 phages | | | | |  |  |  |  |  |
| --- | --- | --- | --- | --- | --- | --- | --- | --- | --- | --- | --- | --- |
| CDS | aa | Predicted/putative function | phiEco32 |  | NJ01 |  | KBNP135 |  | 07-Nov |  | GAP52 |  |
|  |  |  | NC_015464.1 | | JX867715.1 | | NC_018859 | | NC_015938 | | JN882286 | |
|  |  |  | identity | CDS | identity | CDS | identity | CDS | identity | CDS | identity | CDS |
| Late genes | |  |  |  |  |  |  |  |  |  |  |  |
| 1 | 55 |  | 82% over 73% | 1 | 82% over 71% | 12 | 74% | 1 |  |  |  |  |
| 2 | 84 |  | 93% | 4 | 88% over 86% | 14 | 57% over 97%, 3 gaps | 2 | 29% over 85%, 2 gaps | 2 |  |  |
| 3 | 210 |  | 98% over 97% | 5 | 80% over 98%, 2 gaps | 15 | 55% over 61%, 1 gap | 3 |  |  |  |  |
| 4 | 157 |  | 99% | 6 | 99% | 16 | 91% over 89% | 4 | 49% over 71%, 3 gaps | 1 | 48% over 71%, 3 gaps | 1 |
| 5 | 513 | terminase large subunit, COG5565 superfamily | 99% | 7 | 99% | 17 | 93% | 5 | 62% over 97%, 4 gaps | 4 | 60% over 98%, 4 gaps | 5 |
| 6 | 747 | portal protein | 99% | 8 | 99% | 18 | 93% | 6 | 40% over 92%, 6 gaps | 5 | 44% over 48%, 3 gaps | 6 |
| 7 | 78 |  |  |  | 100% | 19 | 82% over 97% | 7 |  |  |  |  |
| 8 | 358 | scaffolding protein | 95%,1 gap | 10 | 94%, 1 gap | 20 | 80% over 98%, 2 gaps | 8 | 41% over 64%, 3 gaps | 7 | 39% over 64%, 2 gaps | 9 |
| 9 | 352 | major head protein | 99% | 11 | 98% | 211 | 94% | 9 | 56% over 99%, 4 gaps | 8 | 55% over 97%, 6 gaps | 10 |
| 10 | 132 | bacterial Ig-like domain | 98% | 12 | 65% over 97% | 22 | 76% over 99% | 10 |  |  |  |  |
| 11 | 250 | conserved protein | 99% | 13 | 98% | 23 | 78% over 99%, 1 gap | 11 | 30% over 88%, 5 gaps | 12 | 31% over 88%, 7 gaps | 15 |
| 12 | 786 | tail fiber protein | 82%, 5 gaps | 14 | 88%, 2 gaps | 24 | 66%, 7 gaps | 12 |  |  |  |  |
| 13 | 723 | tail protein | 67%,13 gaps | 15 | 62%, 18 gaps | 25 | 58%, 15 gaps | 13 |  |  |  |  |
| 14 | 72 | holin | 100% | 16 | 99% | 26 | 75% over 98% | 14 |  |  |  |  |
| 15 | 163 | lysis protein, lysozyme_like superfamily | 99% | 17 | 99% | 271 | 91% | 15 |  |  |  |  |
| 16 | 267 | structural protein, base plate wedge and pin domain | 100% | 18 | 99% | 28 | 82% | 16 | 28%, 10 gaps | 17 | 26%, 10 gaps | 19 |
| 17 | 1005 | bacterial surface protein, big_2 superfamily domain | 99% | 19 | 98% | 29 | 83%, 3 gaps | 17 | 33% over 65%, 15 gaps | 18 | 30% over 51%, 14 gaps | 20 |
| 18 | 322 | tail tip fiber protein, DUF superfamily domain | 100% | 20 | 92%, 1 gap | 301 | 85%, 1 gap | 18 |  |  |  |  |
| 19 | 343 | conserved protein | 97% | 21 | 62%, 2 gaps | 31 | 45% over 96%, 5 gaps | 19 |  |  |  |  |
| 20 | 260 | internal virion protein, lytic transglycosylase domain | 97% | 22 | 97%over 98% | 321 | 85% over 99%, 2 gaps | 20 |  |  |  |  |
| 21 | 350 |  | 97% | 23 | 95% | 33 | 80% over 100%, 1 gap | 21 |  |  |  |  |
| 22 | 322 | DNA injection protein | 99% | 24 | 97% | 34 | 82% | 22 | 30% over 71%, 4 gaps | 20 | 29% over 79%, 4 gaps | 22 |
| 23 | 551 |  | 99% | 25 | 98% over 99%, 3 gaps | 351 | 66%, 5 gaps | 23 |  |  |  |  |
| 24 | 1473 |  | 99% | 26 | 95% | 36 | 79%, 4 gaps | 24 |  |  |  |  |
| Middle genes | |  |  |  |  |  |  |  |  |  |  |  |
| 25 | 53 | conserved protein | 92% | 28 | 94% | 39 |  |  |  |  |  |  |
| 26 | 84 |  | 100% | 29 | 69% |  | 81% | 25 |  |  |  |  |
| 27 | 133 | conserved protein | 94% over 98% | 30 | 98% | 41 | 79% | 26 |  |  |  |  |
| 28 | 59 |  | 100% | 32 | 69% over 98%, 4 gaps | 42 | 81% over 98%, 1 gap | 27 |  |  |  |  |
| 29 | 272 | 5'-3' exonuclease | 100% | 33 | 99% | 43 | 78% | 28 | 49% over 95%, 2 gaps | 45 | 49% over 95%, 2 gaps | 40 |
| 30 | 297 | ATP-binding protein | 97% over 99% | 34 |  |  | 64% over 65%, 2 gaps | 30 |  |  |  |  |
| 31 | 130 | GTP-binding protein | 97% | 35 | 97% | 451 |  |  | 58% over 86% | 37 |  |  |
| 32 | 234 | RNA polymerase ECF sigma factor | 100% | 36 | 99% | 46 | 81% over 99% | 31 | 41% over 28%, 2 gaps | 47 | 28% over 80% | 42 |
| 33 | 146 | Appr-1-p processing enzyme. | 99% | 37 |  |  |  |  |  |  |  |  |
| 34 | 57 | conserved protein | 100% | 38 | 100% | 47 | 84% | 33 |  |  |  |  |
| 35 | 116 | conserved protein | 100% | 39 | 93% | 481 | 80% over 96% | 34 |  |  |  |  |
| 36 | 138 | HNH endonuclease domain (E 2e-19) | |  |  |  |  |  |  |  |  |  |
| 37 | 248 | conserved protein | 99% | 40 | 98% | 49 | 85% | 35 | 44% over 79%, 2 gaps | 48 | 41% over 93%, 4 gaps | 43 |
|  |  |  |  |  |  |  |  |  | 2 gaps |  | 4 gaps |  |
| 38 |  |  |  |  |  |  |  |  |  |  |  |  |
| 39 | 54 |  | 92% over 94% | 42 | 98% | 50 |  |  |  |  |  |  |
| 40 | 167 | serine/threonine protein phosphatase | |  |  |  | 89% over 97% | 36?^1^ | 56% over 95%, 3 gaps | 51 |  |  |
| 41 | 71 |  | 97% | 43 | 97% | 53% 1 | 61% over 98%, 1 gap | 38 | 51% over 95%, 2 gaps | 53 | 69%, 1 gap | 48 |
| 42 | 47 |  | 100% | 44 | 96% | 54 | 53% over 85% | 39 |  |  |  |  |
| 43 | 55 |  | 100% | 45 |  |  | 94% over 96% | 41 |  |  |  |  |
| 44 | 58 |  | 98% | 46 | 95% over 98% | 56 | 89% over 96% | 42 |  |  |  |  |
| 45 | 130 |  | 86% | 47 | 87% | 57 | 77% | 43 | 47% over 79%, 1 gap | 52 |  |  |
| 46 | 58 |  | 98% over 98% | 48 | 95% over 96% | 58 | 60% over 77% | 44 |  |  |  |  |
| 47 | 55 |  | 100% | 49 | 100% | 59 | 56% over 94% | 45 |  |  |  |  |
| 48 | 42 |  | 100% | 50 | 98% | 601 | 45%, 1 gap | 46 |  |  |  |  |
| 49 | 63 | Conserved protein | 100% | 51 | 98% | 61 |  |  |  |  |  |  |
| 50 | 121 |  | 100% | 52 |  |  |  |  |  |  |  |  |
| 51 | 614 | DNA polymerase-like protein | 99% | 53 | 98% | 62 1 | 87% over 91% | 50 | 46% over 98%, 6 gaps | 63 | 48% over 98%, 9 gaps | 51 |
| 52 | 137 |  | 98% | 54 | 83%, 1 gap | 63 | 39%, 3 gaps | 51 |  |  |  |  |
| 53 | 94 |  | 89% | 56 | 81%, 1 gap | 65 | 39% over 95%, 2 gaps | 52 |  |  |  |  |
| 54 | 84 |  | 66%, 2 gaps | 57 | 74%, 1 gap | 66 | 57% over 60%, 1 gap | 53 |  |  |  |  |
| 55 | 97 | 50% identity to hypothetical protein Cronobacter phage GAP31, YP_006986916.1 | | | | | | |  |  |  |  |
| 56 | 104 |  | 100% | 58 | 99% | 67 | 87%, 2 gaps | 54 | 59% over 70% | 68 | 66% over 90% | 54 |
| 57 | 245 | PhoH-like protein:KH, type 1 | 99% over 99% | 60 | 98% | 68?^1^ | 89% over 99%, 1 gap | 55 | 72% over 98%, 2 gaps | 80 |  |  |
| 58 | 61 |  | 97% | 61 |  |  |  |  | 39% over 91% | 76 |  |  |
| 59 | 109 | NAD-dependent DNA ligase | 99% | 62 | 97% over 96% | 72?^1^ | 77% over 94% | 56 |  |  |  |  |
| 60 | 61 |  | 90% | 63 | 93% |  | 90% |  |  |  |  |  |
| 61 | 63 |  |  |  | 100% | 73 |  |  |  |  |  |  |
| 62 | 44 | putative NAD+ diphosphatase | | | 98% over 97% | 74 |  |  |  |  |  |  |
| 63 | 82 |  |  |  |  |  |  |  |  |  |  |  |
| 64 | 218 | thymidylate synthase thyX/thy1 | 99% | 64 | 98% | 75?^1^ | 80% over 98% | 58 | 64% over 97%, 3 gaps | 74 |  |  |
| 65 | 91 | thiol-disulphide isomerase and thioredoxin | 98% | 65 | 98% over 96% | 77 | 78% over 97% | 61 | 41% over 95% | 79 | 41% over 95% | 64 |
| 66 | 59 |  | 100% | 66 | 97% | 80 | 53% | 64 |  |  |  |  |
| 67 | 187 | DNA-binding protein – ferritin-like superfamily | 97% | 67 | 99 over 83% | 81 | 81%, 1 gap | 65 | 42% over 78%, 1 gap | 83 | 39% over 71%, 2 gaps | 62 |
| 68 | 109 | conserved protein | 100% | 68 | 100% | 821 | 93% | 66 | 36% over 74% | 87 | 31% over 73% | 63 |
| Early genes | |  |  |  |  |  |  |  |  |  |  |  |
| 69 | 69 |  | 97% | 69 | 91% | 83 | 41% (3 gaps) | 67 |  |  |  |  |
| 70 | 69 |  | 100% | 70 | 88% | 84 | 48% over 92%, 1 gap | 68 |  |  |  |  |
| 71 | 65 |  | 65% | 71 | 71% | 85 | 38% over 98%, 3 gaps | 67 |  |  |  |  |
| 72 | 68 |  |  |  | 78% | 86 |  |  |  |  |  |  |
| 73 | 177 | dCTP deaminase | 99% | 72 | 99% | 871 | 95% | 70 | 50% over 97%, 1 gap | 81 | 55% over 97% | 65 |
| 74 | 456 | nucleotidyltransferase domain of class II CCA-adding enzyme translation | 85%, 1 gap | 73 | 84%, 1 gap | 88 | 32% over 63%, 5 gaps | 71 |  |  |  |  |
| 75 | 185 | type I DNA polymerase | 99% | 74 | 96% over 98% | 891 | 80% over 97% | 72 | 49% over 94% | 82 | 50% over 96% | 67 |
| 76 | 596 | primase/helicase | 99% | 75 | 100% | 901 | 94% | 73 | 56% over 97%, 4 gaps | 84 | 58% over 96%, 3 gaps | 68 |
| 77 |  |  |  |  |  |  |  |  |  |  |  |  |
| 78 | 139 | conserved YtfP/UPF0131 protein | 98% | 77 | 100% | 92 | 65% over 99% | 75 | 50% over 91% | 86 | 53% over 93% | 86 |
| 79 | 69 | conseved protein | |  | 100% | 93 |  |  | 60% over 98% | 93 |  |  |
| 80 | 61 |  | 95% | 78 | 95% | 94 | 39% | 77 |  |  |  |  |
| 81 | 80 |  | 100% | 79 | 100% | 95 | 80% | 78 |  |  |  |  |
| 82 | 400 | ATP-grasp enzyme | 98% | 80 | 87% | 96 | 59 over 98% | 79 | 41% over 65% | 99 | 44% over 64% | 78 |
| 83 | 74 |  | 100% | 81 | 100% | 97 | 58% over 90% | 80 |  |  |  |  |
| 84 | 77 |  | 100% | 82 | 100% | 98 | 84% | 81 | 40% over 88% | 101 | 38% over 88% | 80 |
| 85 | 677 | glutamine aminotransferase | 99% | 83 | 99% | 99 | 72% | 82 | 35% | 102 | 37% | 81 |
| 86 | 367 | amidoligase enzyme | 98% | 84 | 98% | 100 | 71% | 83 | 44% over 73% | 103 | 43% over 71% | 82 |
| 87 | 205 | conserved protein | 100% | 85 | 98% | 101 | 65% | 84 | 28% over 80% | 104 | 28% over 81% | 83 |
| 88 | 268 | Conserved protein | 99% | 86 | 99% | 102 | 78% over 99% | 85 | 48% over 98% | 105 | 48% over 98% | 84 |
| 89 | 323 |  | 97% | 87 | 96% | 103 | 53% | 86 |  |  |  |  |
| 90 | 399 |  | 96% | 88 | 89% | 104 | 43% | 87 |  |  |  |  |
| 91 | 120 |  | 94% | 89 | 94% | 105 | 59% over 97% | 88 |  |  |  |  |
| 92 | 50 |  | 98% | 90 | 96% | 106 | 33% over 98% | 89 |  |  |  |  |
| 93 | 154 |  | 79% | 92 |  |  |  |  | 30% |  |  |  |
| 94 | 77 | conserved protein | 100% | 93 | 100% | 108 | 89% over 98% | 91 |  |  |  |  |
| 95 | 38 |  | 100% | 94 | 95% | 109 | 68% | 93 |  |  |  |  |
| 96 | 86 |  | 84% over 94% | 95 | 56% over 95% | 110 | 39% over 79% | 95 |  |  |  |  |
| 97 | 81 |  | 67% | 96 | 68% | 111 |  |  |  |  |  |  |
| 98 | 84 |  | 28% over 90% | 97 | 29% over 90% | 112 |  |  |  |  |  |  |
| 99 | 103 | Myb-like DNA binding domain, SANT superfamily | 63% | 99 | 98% over 90% | 113 |  |  | 36% over 98% | 116 |  |  |
| 100 | 43 | conserved phage protein | 81% | 101 | 86% | 115 | 65% over 93%. 2 gaps | 101 |  |  |  |  |
| 101 | 58 | DNA topoisomerase I domain | 91%, 1 gap | 103 | 97%, 1 gap | 1161 |  |  |  |  |  |  |
| 102 | 73 |  | 93% | 104 | 84% | 117 |  | 99 |  |  |  |  |
| 103 | 82 |  | 96% | 105 | 98% | 118 | 38% over 85%, 1 gap | 100 |  |  |  |  |
| 104 | 93 | lipoprotein | 99% | 106 | 71%, 1 gap | 1201 | 67% | 104 |  |  |  |  |
| 105 | 54 |  | 56% | 107 | 46% | 123 | 44% | 105 |  |  |  |  |
| 106 | 83 |  | 96% | 108 | 98% | 124 | 64% over 96% | 111 |  |  |  |  |
| 107 | 74 |  | 99% | 109 | 95% | 125 |  |  |  |  |  |  |
| 108 | 58 |  | 98% | 110 | 98% | 126 |  |  |  |  |  |  |
| 109 | 59 |  | 100% | 111 | 98% | 129 | 63% over 89%, 1 gap | 107 |  |  |  |  |
| 110 | 46 |  | 100% | 112 | 98% | 130 | 63% | 108 | 47% over 69% | 135 |  |  |
| 111 | 72 |  | 99% | 113 | 96% | 131 |  |  |  |  |  |  |
| 112 | 85 |  | 59% | 114 | 55% over 97% | 133 | 29% over 97% | 109 |  |  |  |  |
| 113 | 78 |  | 94% | 115 | 90% |  | 87% |  |  |  |  |  |
| 114 | 78 |  |  |  | 68% | 135 | 39% over 97% | 109 |  |  |  |  |
| 115 | 99 |  | 93% | 116 | 98% over 58% | 136 | 46% over 97% | 113 |  |  |  |  |
| 116 | 129 |  | 100% | 117 | 83% | 137/01 |  |  |  |  |  |  |
| 117 | 147 |  | 99% | 120 | 98% | 1 | 99% | 115 |  |  |  |  |
| 118 | 69 |  | 80% | 121 | 91% | 2 |  |  |  |  |  |  |
| 119 | 72 |  | 99% | 123 | 92% | 4 | 100% | 117 |  |  |  |  |
| 120 | 299 |  | 98% | 124 | 96% | 5 | 96% | 118 |  |  |  |  |
| 121 | 121 |  | 88% | 125 | 87% | 7 |  |  |  |  |  |  |
| 122 | 86 |  | 92% | 126 |  |  |  |  |  |  |  |  |
| 123 | 73 |  |  |  |  |  |  |  |  |  |  |  |
| 124 | 87 |  | 77% | 127 | 62% over 94% | 9 | 78% | 119 |  |  |  |  |
| 125 | 290 |  | 98% | 128 | 83% over 55% | 10 | 83% over 55% | 120 |  |  |  |  |
