# Supplementary material for: Genomic, Proteomic, Morphological, and Phylogenetic Analyses of vB_EcoP_SU10, a Podoviridae Phage with C3 Morphology
Source: PLoS One. 2014 Dec 31;9(12):e116294. doi: 10.1371/journal.pone.0116294 (PMC4281155; doi:10.1371/journal.pone.0116294)
Supplement: S2 Table — Early and middle transcription promoter and terminator sequences and their location in the genome of SU10. (DOCX) [file pone.0116294.s003.docx]

**Table S2**. Early and middle transcription promoter and terminator sequences and their location in the genome of SU10.

Early promoters Locations

76677-ttgacagccttttcctttaatatatagtttgcgagtg upstream of CDS125

-35 -10

73996-ttgacaccgaaaacagcctgatatatagtgagcatagt between CDS120 and CDS121

-35 -10

73668-ttgacaagctaacacgatgtgttaagctgtaaatg between CDS120 and CDS121

-35 -10 alt -10?

73511-ttgacagcgaaaacaacatgattcataatgagcaca between CDS120 and CDS121

-35 -10

Middle promoters

47227 ttggtagtagatatgataactctcaaatgtatatagagtac between CDS68 and CDS69

-35 -10

43288 acctgtatggacagatatagacaaatattatcggttgtt upstream of CDS32

-35 -10

43039 acttataatgtatactccttagtgtacactgatatattaag between CDS56 and CDS57

36247 aagcgtaatgtatactatacataaccgctcaaagagagcaa between CDS37 and CDS38

Late promoters

25622 ttctataatgtatatagggggtaggacatttttatttat between CDS23 and CDS24

8988 gtgtgtaatgtatattctttgtctacatataacaaggag between CDS10 and CDS11

1480 gttgataatgtagaaggagtataaaatgtttggagaaaa between CDS3 and CDS4

Promoters at the start of the genome:

3679-taaagtaagggggaggtttgtcctcccttcttttatttaagctatcaaccgagggagagaatcgatg

Between CDS5 and CDS6, contains a potential sigma-70 promoter, but lacks consensus for a late promoter

1980-taaaggaaacagggagctgtcgaagatcatctgattaacaatctaaggattgttatagttttattgccacgatgttcttttgcagtgtacccttgtagtggagaggggaggttctttgacgaaggggactttcccttttctatttttaaactggaggaagtatg

Between CDS4 and CDS5, contains a potential sigma-70 promoter, but lacks consensus for a late promoter

177-acacgcgggaagcggctagccccactcagtcaaattgaaattttaaacacttatatataaggttgaaagagaaattatg

Upstream region of CDS1, contains a potential sigma-70 promoter, but lacks consensus for a late promoter

Terminators:

3682-agtaagggggaggtttgtcctcccttcttttatttaa

8934-aactagaccctgccttcgggcggggttttttcgtttt

30143-gaaacaaataaagccctcttggatttctcctcgagggcttttctttt

74441-tcctcctataagcctcactatgaggcttttttcttttct
